# Flavones from *Erythrina falcata* are modulators of fear memory

**DOI:** 10.1186/1472-6882-14-288

**Published:** 2014-08-05

**Authors:** Daniela Rodrigues de Oliveira, Cláudia R Zamberlam, Renan Barreta Gaiardo, Gizelda Maia Rêgo, Janete M Cerutti, Alberto J Cavalheiro, Suzete M Cerutti

**Affiliations:** Department of Biological Science, Behavior Pharmacology and Ethnopharmacology Laboratory, Universidade Federal de Sao Paulo, Sao Paulo, SP Brazil; Department of Morphology and Genetics, Genetic Bases of Thyroid Tumor Laboratory, Division of Genetics, Universidade Federal de Sao Paulo, Sao Paulo, SP Brazil; Department of Forestry Colombo, Brazilian Agricultural Research Corporation (EMBRAPA), Paraná, Brazil; Institute of Chemistry, Nuclei of Bioassay, Biosynthesis and Ecophysiology of Natural Products (NuBBE), State University of Sao Paulo, UNESP, Araraquara, SP Brazil

**Keywords:** Flavone, Acquisition, Extinction, Fear conditioning, *Erythrina falcata* (Fabaceae), HPLC-ESI/MS^n^

## Abstract

**Background:**

Flavonoids, which have been identified in a variety of plants, have been demonstrated to elicit beneficial effects on memory. Some studies have reported that flavonoids derived from *Erythrina* plants can provide such beneficial effects on memory. The aim of this study was to identify the flavonoids present in the stem bark crude extract of *Erythrina falcata* (CE) and to perform a bioactivity-guided study on conditioned fear memory.

**Methods:**

The secondary metabolites of CE were identified by high performance liquid chromatography combined with a diode array detector, electrospray ionization tandem mass spectrometry (HPLC-DAD-ESI/MS^n^) and nuclear magnetic resonance (NMR). The buthanolic fraction (BuF) was obtained by partitioning. Subfractions from BuF (BuF1 – BuF6) and fraction flavonoidic (FfA and FfB) were obtained by flash chromatography. The BuF3 and BuF4 fractions were used for the isolation of flavonoids, which was performed using HPLC-PAD. The isolated substances were quantified by HPLC-DAD and their structures were confirmed by nuclear magnetic resonance (NMR). The activities of CE and the subfractions were monitored using a one-trial, step-down inhibitory avoidance (IA) task to identify the effects of these substances on the acquisition and extinction of conditioned fear in rats.

**Results:**

Six subclasses of flavonoids were identified for the first time in CE. According to our behavioral data, CE, BuF, BuF3 and BuF4, the flavonoidic fractions, vitexin, isovitexin and 6-C-glycoside-diosmetin improved the acquisition of fear memory. Rats treated with BuF, BuF3 and BuF4 were particularly resistant to extinction. Nevertheless, rats treated with FfA and FfB, vitexin, isovitexin and 6-C-glycoside-diosmetin exhibited gradual reduction in conditioned fear response during the extinction retest session, which was measured at 48 to 480 h after conditioning.

**Conclusions:**

Our results demonstrate that vitexin, isovitexin and diosmetin-6-C-glucoside and flavonoidic fractions resulted in a significant retention of fear memory but did not prevent the extinction of fear memory. These results further substantiate that the treatment with pure flavonoids or flavanoid-rich fractions might represent potential therapeutic approaches for the treatment of neurocognitive disorders, improvement of memory acquisition and spontaneous recovery of fear.

## Background

Despite remarkable progress that has been made on the understanding of how the brain acquires, stores, and retrieves information, modest progress has been made in treating cognitive deficit. The loss of memory can causes a range of disabilities, which can significantly impact daily performance in terms of simple tasks and difficulty in making choices, potentially resulting in poor judgment or socially inappropriate behavior. Therefore, the development of new therapeutic drugs for the treatment of cognitive impairment remains a major focus of neuroscience.

Accumulating evidence has shown that the herbs represent a promising source of novel therapeutic agents. Recent studies from our laboratory have shown that short- and long-term treatment with a standardized, flavanoid-rich extract of *Ginkgo biloba* (EGb) can improve conditioned-fear memory. However, unlike diazepam, which is traditionally used to reduce anxiety, the acute treatment with EGb did not cause deficits in the acquisition of a conditioned emotional response (CER) [1, 2]. Conversely, *Erythrina velutina*, a plant that is rich in alkaloids and flavonoids, is widely used for anxiolytic purposes. At low doses, it impairs the acquisition of fear conditioning in inhibitory avoidance task, whereas at high doses, it predominantly elicits sedative and neuromuscular blocking effects [3].

*Erythrina* (Fabaceae family) is a genus of approximately 130 species from tropical and subtropical regions worldwide. It is widely distributed in south and southeastern Brazil, north Argentine and Paraguay, south Bolivia and Peru [4]. *Erythrina* species are known to produce alkaloids [5–9], terpenes [10] and flavonoids [7, 8, 10–15], and their stem bark and leaves are commonly used to make teas (infusion or decoction) that are believed that to exhibit tranquilizing and anti-anxiety properties [16, 17]. Based on their common use in folk medicine, most research on the genus has involved isolating and characterizing their alkaloid constituents. Information regarding the flavonoids derived from *Erythrina* species, as well as their effects on the central nervous system, remains limited. Previous studies have suggested that phytochemicals, particularly flavonoids, might modulate protein and lipid kinase signaling pathways [1, 2, 18–21]. Further, polyphenols have been associated with a reduced risk of the development of dementia [22].

Flavonoids represent a large family of compounds that are synthesized by plants and that comprise 15 carbons with two aromatic rings connected by a three-carbon bridge (C_6_-C_3_-C_6_). Naturally occurring anxiolytic flavonoids were first described in 1990s [23–26]. Flavonoids have been investigated using *in vivo* models, where their effects on the central nervous system (CNS) have established. More recently their antioxidant activity, which are attributed to their ability to inhibit the production of free radicals, and their neuroprotective effects were found to be related. Moreover, there has been intense interest in the potential of flavonoids to modulate neuronal function and to improve memory [22], as well as their activation of microglia and astrocytes that might shape synaptic plasticity [27].

Hence, the isolation and characterization of the active constituents of crude plant extracts can offer great advantages and provide opportunities for new drug discoveries. In this sense, it is important elucidate which constituents contained within the crude extract of the *Erythrina falcata* (*E.falcata*) contribute directly or indirectly to its bioactivity. To elucidate the effects of acute treatment with stem bark crude extract of *Erythrina falcata* (CE) or of its secondary metabolites on conditioned-fear memory, we employed a one-way inhibitory avoidance learning (IA) task, which has been previously used to assess fear memory in rodents that are sensitive to anxiolytic drugs.

Memory is considered to be a process that includes acquisition, consolidation and retrieval. In fear-conditioned memory, acquisition refers to the process in which an association between a neutral conditional stimulus (CS) and an aversive unconditional stimulus (US) is formed through the pairing of the CS with the US. Consolidation refers to a progressive, time-dependent stabilization process, which transforms short-term memories into relatively permanent, long-term memories. Memory retrieval is characterized as the subsequent recovery of events or information from the past, which have been previously stored within specific neural systems. During the retrieval process, two opposite processes, reconsolidation and extinct, are initiated, and the memories become labile and can be affected by pharmacological manipulations at different stages of this process [28]. The effects of flavonoid-rich plants and pure flavonoids on memory have been shown in rodents.

In this study, a monitored fractionation study was used to identify the effects of isolated pure flavonoids, as well as of flavonoidic fractions, from crude extracts of *E. falcata* on the acquisition and extinction of conditioned fear in rats.

## Methods

### The extraction, fractionation, isolation and characterization of bioactive compounds from crude extracts of *E. falcata*

#### Chemicals Reagents

Methanol (HPLC grade) and dimethyl sulfoxide-d_9_ (99.8%) were obtained from Merck (Darmstadt, Germany). Formic acid, ethanol and *n*-buthanol were obtained from Synth (Diadema, Brazil). Water was produced in our laboratory (Milli-Q Water Purification System, Millipore). Vitexin and isovitexin standards (99.99%) were purchased from Sigma-Aldrich (São Paulo, Brazil).

### Plant material

The stem bark of *E. falcata* plants was collected in October 2009 in Colombo, Paraná, Brazil. The samples were registered as number 173 and stored in the herbarium of the National Center Research of Forestry – CNPF, at the Brazilian Agricultural Research Corporation (EMBRAPA), Brazil.

### The preparation of the extract and the monitored fractionation and identification of its constituents

**Figure 1:**
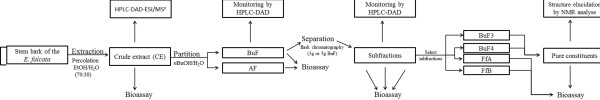
**A schematic of the process used for the preparation of** ***Erythrina falcata*** **extracts and subsequent bioactivity-guided fractionation.**

The stem bark (1 Kg) was extracted by percolation with EtOH/H_2_O (70:30) at room temperature for 1 month. The extract was concentrated into a small volume using a rotatory evaporator and then freeze-dried to produce a crude extract (CE, 100 g). The CE (50 g) was suspended in H_2_O/MeOH (2:1) and partitioned using *n*BuOH (*n-*buthanol) (3 × 650 mL) and water, producing a buthanolic total fraction (BuF) and an aqueous fraction (AF). These fractions were concentrated into a small volume at 37°C using a rotatory evaporator and then freeze-dried, yielding 13.48 and 32.68 g of BuF and AF, respectively. Part of the BuF (3.0 g) was further fractionated by flash chromatography on a C18 (40 μm, APD, 60 Å, J.T. Baker, USA) column (2.5 cm × 40 cm), using step gradient elution of H_2_O to MeOH (1:0; 8:2; 6:4; 4:6; 2:8; 0:1). Six fractions (90 mL each) were collected (BuF1- BuF6). Another portion of BuF (5 g) was fractionated again by flash chromatography on a C18 (40 μm, APD, 60 Å, J.T. Baker, USA) columm (4.5 cm × 40 cm), under the same conditions, subsequently yielding 15 fractions (50 mL each, Ff1A-Ff1C, Ff2A-Ff2C, Ff3A-Ff3C, Ff4A-Ff4C and Ff5A-Ff5C) to produce a flavonoid-enriched fraction (Figure 1).

**Figure 2:**
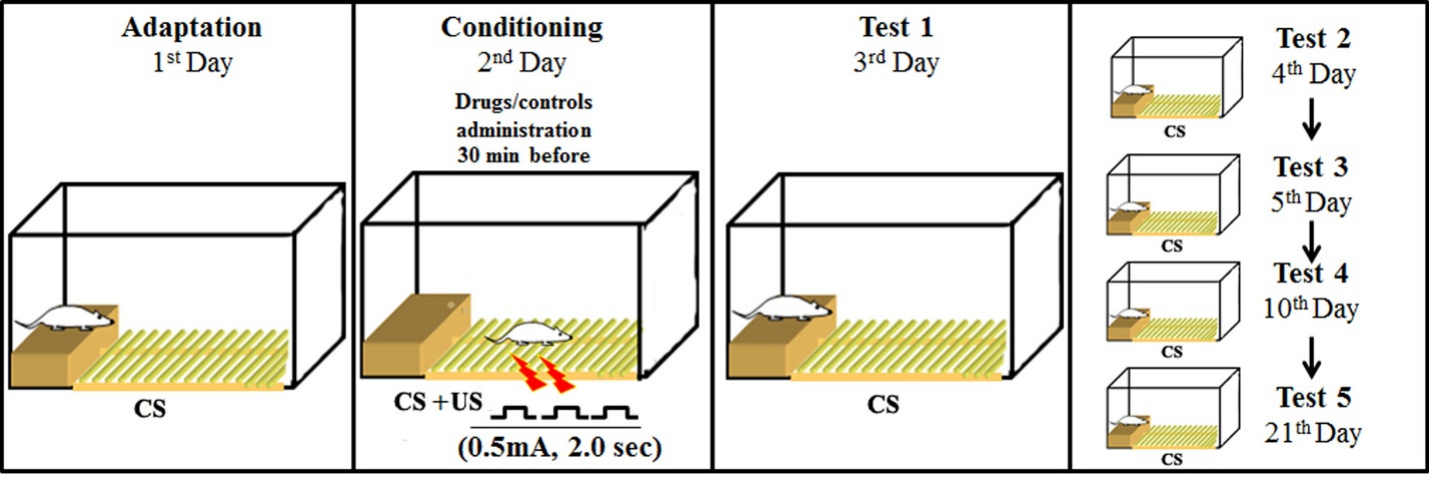
**A schematic of the experimental procedure for the one-trial, step-down avoidance inhibitory task (IA) and drug administration schedule, which was the same for all of the animal groups except for the CS group.** The rats evaluated by the IA task were subjected to one training session (a 2-s, 0.5-mA footshock (US) associated with a context (CS)). The animals were subjected to five retention test sessions (trials) of IA tasks, and the step-down latencies were measured. No footshock was administered in the test sessions. The interval between trials T1, T2 and T3 was 24 h. T4 and T5 comprised 240- and 480-h intervals, respectively.

HPLC-DAD analysis was performed using a Shimadzu apparatus equipped with a DGU-20A_5_ degasser, two LC-20AT pumps, a CBM-20A controller, a SIL-20A_3_ autosampler, a CT0-20A oven column and a SPD-M20A diode array detector (DAD, Shimadzu (Kyoto, Japan). The studies were conducted on a C18 Luna® column (250 mm × 4.60 mm, 5 μm) (Phenomenex, Torrance, CA, USA). The mobile phase consisted of 0.1% aqueous formic acid (A) and methanol (B). A gradient of A/B (95:5 to 1:1, v/v in 50 min) was used. UV spectra were achieved from 200–400 nm, and the chromatogram registered at 254 nm. The flow rate was maintained at 1.0 mL.min^−1^, sample concentration was 1 mg.mL^−1^ and the injection volume was 20 μL. Analysis of the data was performed using LC Solution® 1.23 SP1 software (Shimadzu, Kyoto, Japan). The classes of the compounds were identified according to their UV spectra and retention time. After the chromatographic profile analyses, all of the CE, BuF, subfractions (BuF1-Buf6), FfA and FfB samples were subjected to evaluation of their bioactivities using a one-trial, step-down avoidance inhibitory (IA) test (Figure 2).

Our behavioral results indicate that the BuF3 and BuF4 subfractions can modulate the retention of fear memory. Consequently, fractions BuF3 (0.45 g) and BuF4 (0.31 g) were subjected to the further isolation of flavonoids, which was performed using a preparative HPLC-PAD (CBM-20A, Shimadzu, Kyoto, Japan) equipped with a DGU-20A_5_ degasser, two LC-6 AD pumps, a CBM-20A controller, a SIL-10AF autosampler_,_ a SPD-M20A photodiode array detector (PAD) and a C18 Luna® column (Phenomenex, Torrance, CA, USA) (150 mm × 21.20 mm, 5 μm, 100 Å). The mobile phase consisted of 0.1% aqueous formic acid (A) and methanol (B). An isocratic elution method of A/B was used for BuF3 (70:30, v/v) and for BuF4 (64:36, v/v), both at a flow-rate of 5 mL.min^−1^. The sample concentrations were 10 mg.mL^−1^, injection volumes were 2 mL and detection was monitored at 254 nm. The compounds were collected according to their UV spectrum profiles and t_R_ (retention time) (Figure 1C). BuF3 yielded compound 1 (11.8 mg, t_R_ 19.36 min) and BuF4 yielded compounds 3 (10.8 mg, t_R_ 22.76 min), 4 (8.1 mg, t_R_ 23.66 min) and 5 (6.6 mg, t_R_ 23.80 min). The activity of these compounds was also evaluated.

The identification of the isolated compounds was based on mass spectrometry (MS) and nuclear magnetic resonance (NMR) analyses. The ^1^H spectra were recorded using a Varian INOVA 500 spectrometer with dimethylsulfoxide-d_9_ as a solvent. The ^1^H NMR spectra at 500 MHz and the ^13^C NMR spectra at 250 MHz were expressed as δ ppm, with reference to TMS, and the coupling constants (*J*) were expressed as Hertz (Hz). The results obtained were compared with NMR spectra that have been previously published.

The CE was additionally analyzed by online high performance liquid chromatography combined with electrospray ionization tandem mass spectrometry (HPLC-ESI/MS^n^) using a Thermo LCQ Fleet System mass spectrometer (Thermo Scientific, San Diego, CA, USA) equipped with an electrospray interface (ESI) and HPLC (model Accela, Thermo Scientific). CE separation was performed using a Luna® C18 column (250 mm × 4.60 mm; Phenomenex, Torrance, CA, USA) at room temperature. The mobile phase consisted of 0.1% aqueous formic acid water (A) and methanol (B). A gradient elution method of A/B (95:5 to 1:1, v/v) was used over 50 min. Ultraviolet (DAD) detection was performed at 254 nm, the flow rate was kept at 0.8 mL/min, the sample concentrations were 1 mg.mL^−1^ and the injection volumes were 10 μL. The column effluents were analyzed by ESIMS in negative ion mode in the mass-to-charge ratio (*m/z)* range of 50–2000, with a scan time of 0.3 s in the centroid mode. The ESI conditions were as follows: nebulizer gas (nitrogen), 30 psi; drying gas, 60 L.min^−1^; drying temperature, 280°C; capillary voltage, 4000 V; nitrogen as collision gas and collision energy at 1 V. The data were acquired in MS and MS^n^ scanning modes. The CE was dissolved in H_2_O:MeOH (1:1 v/v) and infused directly via a syringe pump (flow rate 5 μL.min^−1^) in the ESI source. The data were analyzed using Xcalibur 2.0 Software® (Thermo Scientific).

The nomenclature ascribed to the fragmented ions for the glycoconjugates were previously proposed by Domon and Costello [29].

### Quantitative analysis of flavonoids

The flavonoids were quantified by HPLC-DAD on a Luna® C18 column (Phenomenex, Torrance, CA, USA) (250 mm × 4.60 mm, 5 μm). The mobile phase consisted of 0.1% aqueous formic acid (A) and methanol (B). An isocratic elution method of A/B (64:36, v/v) was used over 50 min. Detection was performed at 254 nm, the flow rate was maintained at 1 mL.min^−1^, sample concentrations were 1 mg.mL^−1^ and the injection volumes were 10 μL. Analytical curves were constructed using solutions of standards from compounds 1, 3, 4 and 5 (1 mg.mL^−1^of each compound in methanol/water 80:20), reaching concentrations within a range of 100.0 to 1000 mg.mL^−1^. Sample peak areas were integrated at 254 nm. All of the procedures were performed in triplicate.

### Monitored fractionation by one-trial step-down inhibitory avoidance (IA) task

#### Subjects

A total of the 200 adult male *Wistar* rats (±250-300 g) with 3 month age were obtained from the CEDEME (Center for Development of Experimental Medicine and Biology, Federal University of Sao Paulo, SP, Brazil). The rats were housed five animals/cages. For 15 days, the animals had free access to food and water under a 12 h:12 h dark:light cycle (lights on at 6:00 to 8:00 h.) with controlled temperatures (21°C ± 2°C) and relative humidity (53 ± 2). These conditions were maintained during the entire experimental period. One minute prior to each experimental session, each rat was placed into an individual cage for transportation to the testing room. All of the procedures for the manipulation of animals were consistent with the Ethical Principles in Animal Research adopted by the Brazilian College for Animal Experimentation (COBEA) and as suggested by the APA Guidelines for Ethical Conduct in the Care and Use of Animals. Filed under number of 0819/10, Research Ethics Committee.

### Experimental protocol

The rats were randomly assigned into 20 groups (n = 10/group) as follows: conditional stimulus group, no-footshock group (CS), negative control (12% Tween®80), positive control (4 mg.Kg^−1^ Diazepam), crude extract of *Erythrina falcata* (CE) (250 mg.Kg^−1^CE and 500 mg.Kg^−1^CE), BuF (145 mg.Kg^−1^), subfractions (20 mg.Kg^−1^BuF1, 16 mg.Kg^−1^ BuF2, 21 mg.Kg^−1^BuF3, 45 mg.Kg^−1^BuF4, 13 mg.Kg^−1^ BuF5 and 27 mg.Kg^−1^ BuF6), vicenin-2 (0.1 mg.Kg^−1^, 0.3 mg.Kg^−1^, 1.0 mg.Kg^−1^ and 10.0 mg.Kg^−1^), vitexin (0.1 mg.Kg^−1^ and 0.25 mg.Kg^−1^), isovitexin (0.1 mg.Kg^−1^ and 0.25 mg.Kg^−1^), 0.1 mg.Kg^−1^ 6-C-glycoside-diosmetin, fraction flavonoidic/Ff (0.65 mg.Kg^−1^ FfB and 0.90 mg.Kg^−1^ FfA).

### Systemic administration

All of the substances were administered orally via gastric tube to ensure complete administration at 30 min before conditioning (training session, Tr). All of the drugs were dissolved in a 12% Tween®80 solution. Diazepam was purchased from Hoffman-La Roche (Brazil), and the dose of Diazepam used in this study was based on the earlier studies conducted in our laboratory [1, 2]. The dose of CE used in this study was based on earlier studies conducted in our laboratory (manuscript in preparation). The buthanolic fraction (BuF), subfractions (BuF1-BuF6), vicenin-2, vitexin, isovitexin, 6-C-glycoside-diosmetin, and the flavonoid fractions FfA and FfB were administered at a rate of 500 mg.Kg^−1^ CE. The CE, subfractions and 12% Tween®80 were administered at 30 min prior to the training session.

### One-trial, step-down avoidance inhibitory

#### Behavior apparatus

The one-trial, step-down avoidance inhibitory (IA) apparatus consisted of a white wood box (50 × 25 × 25 cm), the floor of which consisted of parallel 1.0-mm diameter stainless steel bars spaced at 1.0 cm apart. The left end of the floor was covered by a 7-cm wide, 2.5-cm high wood platform [30–32].

### Behavioral procedure

The rats were fear-conditioned in an IA chamber. The animals were subjected to one training session (Tr) and 5 test sessions (T1, T2, T3, T4 and T5). All of the groups were subjected to treatments with different substances prior to the Tr session as described below, with the exception of the CS group. After each experimental session, the animals were returned to their home cages (Figure 2).

### Adaptation session (1st day)

The animals were gently placed onto the platform facing the left rear corner of the training box, and during a 180- sec period, the animals explored the box (conditioned stimulus - CS). After this period, the animals were returned to their home cages.

### Training session (2nd day)

The rats were placed onto the platform as during adaptation session. When they stepped down and placed all four paws onto the grid, they received a 0.5-mA, 2-s scrambled foot shock (unconditioned stimulus - US). The latency to step-down was measured. After the footshock, the animals were returned to their home cage. This procedure was performed on all of the animals, except for the CS group, which did not receive a footshock. The rats were then tested for retention 24, 48, 72, 240 and 480 h later.

### Test session/ acquisition/ T1 (3rd day)

The rats were tested for retention of fear memory at 24 h (T1) and the step-down latency was used as a measure of retention (to a maximum of 300 s). During the test sessions, no footshocks were administered.

### Test sessions/extinction T2-T5 (4th, 5th, 10th and 21st days)

The animals were subjected to five retention test session (trials) in an IA task, and step-down latencies were measured. No footshock was administered during these tests sessions. The interval between trials T1, T2 and T3 was 24 h. The endpoints of T4 and T5 were designated as 240 and 480 h, respectively, after training.

### Data analysis and statistical procedures

For the Training (Tr) and Test 1 (T1) sessions, the data are reported as mean values (±SEM) of latency to step-down and were analyzed using one-way analysis of variance (ANOVA) followed by Bonferroni’s Multiple Comparison Test. For each rat was measured by differences in test-training session latencies (dL-mean), where dL-mean = T-Tr latency. Comparisons among dL-mean values were analyzed by two-way analysis of variance (ANOVA) followed by Dunnett’s Multiple Comparison. To test the effects of each group and session, as well as the interaction between them, two fixed factors (group and session), one random factor (rat) and repeated measurement of the sessions were considered. All of the statistical analyses were performed using the GraphPad Prism statistics program (Graph Pad 6.0 Software®, San Diego, CA, USA). Differences of the *P* < 0.05 level were considered statistically significant.

## Results

### Identification of the secondary metabolites from crude extract of *E. falcata*

**Figure 3:**
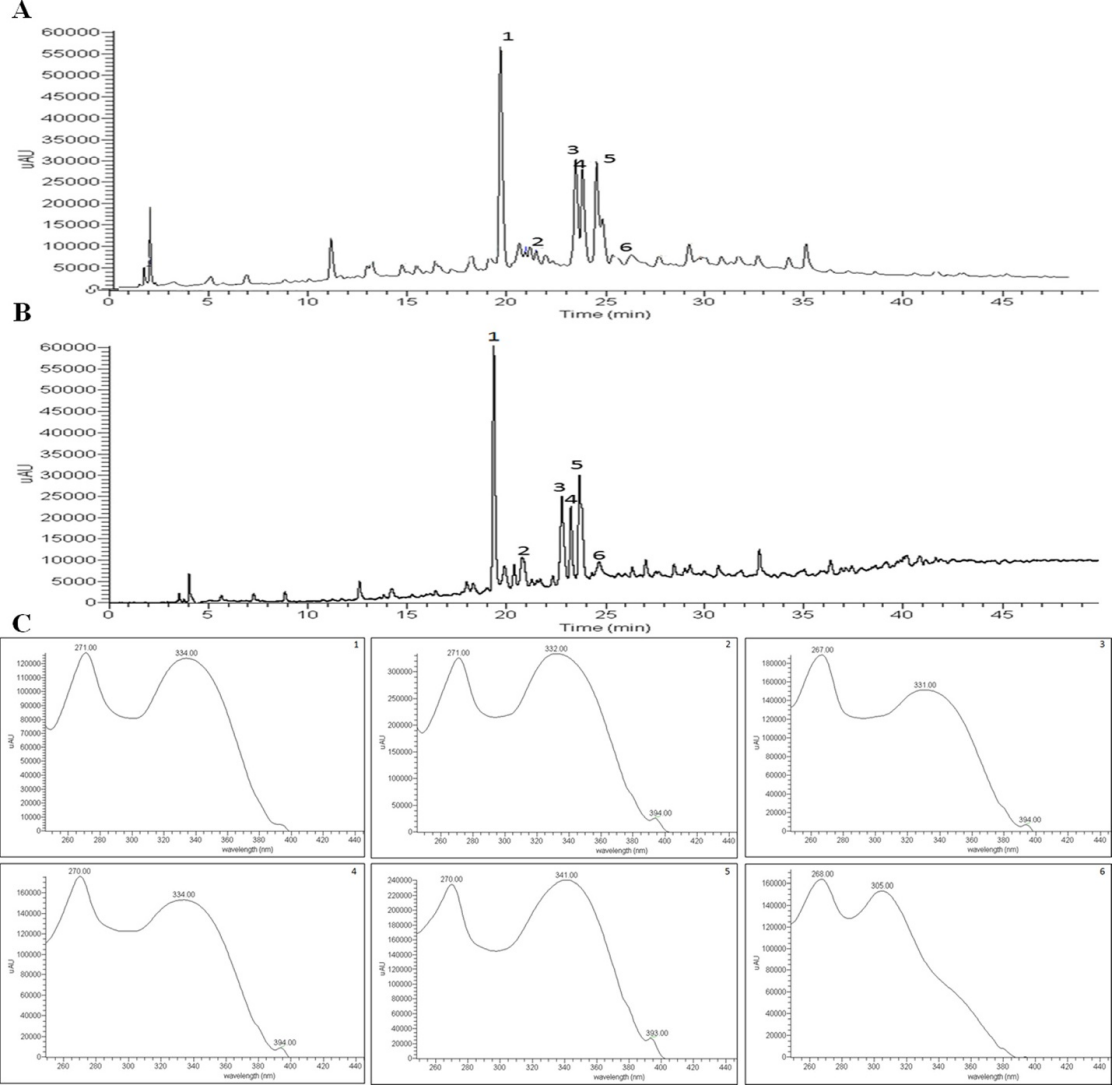
**HPLC-DAD-ESI/MS** ^**n.**^ **analysis of crude extracts (CEs) from the roots of** ***Erythrina falcata*** **using a C18 Luna column (A, B).** The chromatogram was recorded at 254 nm **(A)** and the TIC was in negative mode **(B)**. The UV spectra of compounds 1–6 **(C)**.

The chromatographic profiles of the crude extract of *E. falcata* (CE) stem bark recorded at 254 nm are shown in Figure 3A. The HPLC/UV trace revealed 4 major peaks and several minor peaks. Structural elucidation was based on a combination of UV, NMR and MS data.

**Figure 4:**
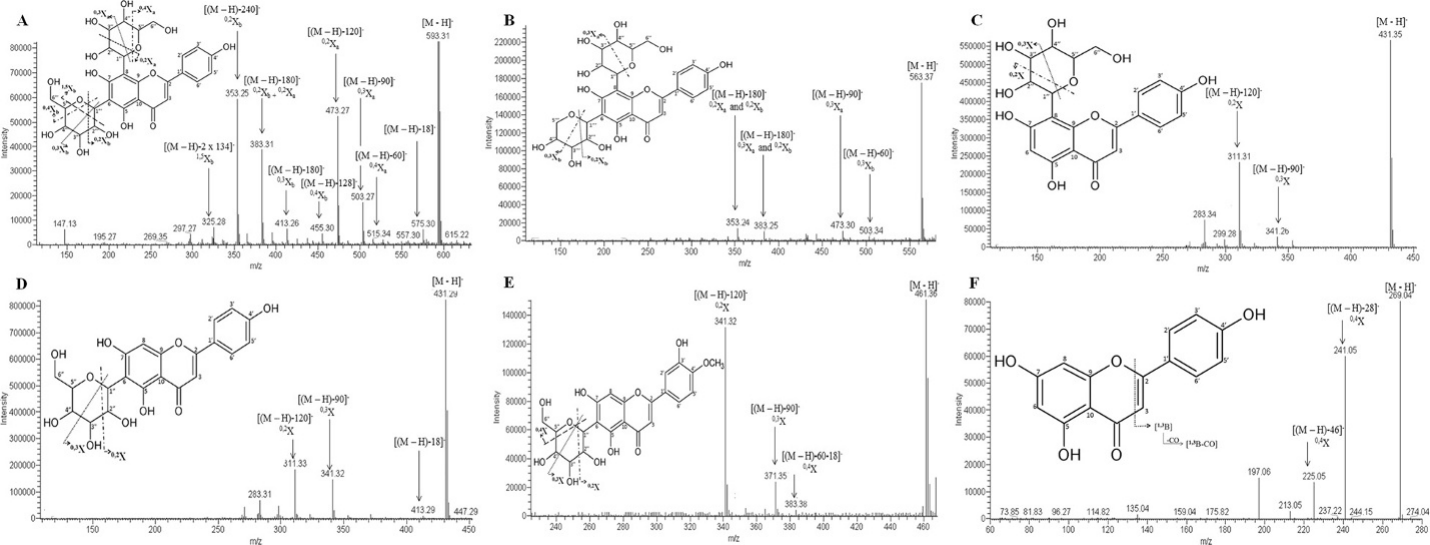
**MS** ^**2**^ **spectra of deprotonated molecules of crude extracts (CEs) from the roots of** ***Erythrina falcata*** **using a C18 Luna column as follows: (A) vicenin-2 [M-H]** ^**−**^ **at** ***m/z*** **593 (1), (B) vicenin-1 [M-H]** ^**−**^ **at** ***m/z*** **563 (2), (C) vitexin [M-H]** ^**−**^ **at** ***m/z*** **431 (3), (D) isovitexin [M-H]** ^**−**^ **at** ***m/z*** **431 (4), (E) 6-C-glycoside diosmetin [M-H]** ^**−**^ **at** ***m/z*** **461 (5) and (F) apigenin [M-H]** ^**−**^ **at** ***m/z*** **269 (6).** Fragment ions used as structural markers are indicated by arrows.

The chromatographic and spectroscopic data of the major and minor peaks of the CE are summarized in Figure 3B, and their structures/fragmentation dates are shown in Figure 4. The UV spectra obtained from the main peaks (1 to 6) observed in the HPLC-DAD analysis reflected a predominance of flavones within these extracts (Figure 3C). The molecular masses of the compounds examined were obtained from the MS data, and these identifications were based on the quasimolecular ion [M-H]^−^ and MS^2^ spectra. The assignment of the NMR signals was based on the published chemical shift values.

Compound 1 exhibits a UV spectrum (λ_max_ = 334, 271 nm, Figure 3C1) that is characteristic of a flavone skeleton [33] and exhibited a quasimolecular ion ([M-H]^−^) at *m/z* 593, yielding secondary fragments at *m/z* 575([M-H]^−^ -18), *m/z* 515 ([^0,4^X_a_]^−^; [M-H]^−^ -60), *m/z* 503 ([^0,3^X_a_]^−^; [M-H]^−^ -90), *m/z* 473 ([^0,2^X_a_]^−^; [M-H]^−^ -120), *m/z* 455 ([^0,4^X_b_]^−^; [M-H]^−^ -128), *m/z* 413 ([^0,3^X_b_]^−^; [M-H]^−^ -180), *m/z* 383 ([^0,4^X_b +_^0,2^X_a_]^−^; [M-H]^−^ -180), *m/z* 353 ([^0,2^X_b_]^−^; [M-H]^−^ -240), and *m/z* 325 ([^1,5^X_b_]^−^; [M-H]^−^ 2x 134) (Figure 4A). This fragmentation pattern is consistent with a 6,8-C-dihexoseflavone and molecular formula C_27_H_30_O_15_. This compound was isolated (Figure 4A) as white crystals. Its ^1^H NMR spectrum (Table 1) in (DMSO-d6) exhibited one aromatic singlet at δ 6.78, assigned to H-3, indicating that the aglycone moiety had substituted C-6 and C-8. The broad singlet at δ 13.7 was attributed to the -OH groups, which form hydrogen bonds. The signal at 6.91 (d, J = 8.0 Hz) and 8.02 (d, J = 8.0 Hz) indicated that ring B contains a *para*-hydroxy group (4′-OH). The carbon signals belonged to the two sugars, confirming that this compound is C-diglucoside flavone. The sites of the sugar linkage to the aglycone were considered to be at positions C-6 and C-8 because the C-8 and C-6 signals appeared at δ 105.26 and 107.58, respectively. This finding was further confirmed by the absence of H-6 and H-8 in the ^1^H NMR spectrum. The spectral data (Table 1) obtained were similar to the reported ones [34, 35], confirming the identification of compound 1 as 6,8-di-*C*-β-*D*-glucopyranoside (vicenin-2).

**Table 1:** **NMR Data for Compounds 1, 3–5 (DMSO-d6, 500 MHz)**

Position	1		3		4		5	
	^1^H NMR (δ _H_)	^13^C NMR (δ _C_)	^1^H NMR (δ _H_)	^13^C NMR (δ _C_)	^1^H NMR (δ _H_)	^13^C NMR (δ _C_)	^1^H NMR (δ _H_)	^13^C NMR (δ _C_)
**2**	-----------	163.9	-----------	-----------	-----------	163.3	-----------	163.3
**3**	6.78 [1H, s]	102.5	6.77 [1H, s]	-----------	6.75 [1H, s]	103.8	6.86 [1H, s]	103.1
**4**	-----------	182.2	-----------	-----------	-----------	182.4	-----------	181.7
**5**	-----------	161.2	13.16 [OH, s]	-----------	13.54 [OH, s]	161.0	8.37 [OH, s]	161.5
**6**	-----------	107.6	6.26 [1H, s]	-----------	-----------	109.2	-----------	-----------
**7**	-----------	161.3	-----------	-----------	-----------	163.9	-----------	163.6
**8**	-----------	105.3	-----------	-----------	6.49 [1H, s]	94.32	6.51 [1H, s]	93.4
**9**	-----------	155.1	-----------	-----------	-----------	-----------	-----------	-----------
**1′**	-----------	121.5	-----------	-----------	-----------	121.4	-----------	123.0
**2′ , 6′**	8.02 [2H, d, (6.5)]	129,0	8.03 [2H, d,(10.0)]	-----------	7.91 [2H, d, (8.5)]	128.9	7.55 [2H, d, (8.0)]	109.8
**3′ , 5′**	6.91 [2H, d, (8.5)]	115.9	6.89 [2H, d, (10.0)]	-----------	6.92 [2H, d, (9.0)]	116.5	6.94 [2H, d, (8.0)]	115.4
**4′**	-----------	161.3	-----------	-----------	-----------	161.2	3.89 [OCH3]	55.6
**1″**	4.71 [1H, d, (9)]	73.4	4.69 [1H, d, (9.5)]	-----------	4.59 [1H, d, (10.0)]	73.6	4.60 [1H, d, (9.5)]	72.9
**2″**	3.38 - 3.80	71.0	3.83 – 3.52	-----------	3.13 -3.89	71.1	3.15- 3.89	69.2
**3″**	3.38 - 3.80	78.8	3.83 – 3.52	-----------	3.13 -3.89	61.9	3.15- 3.89	61
**4″**	3.38 - 3.80	69.1	3.83 – 3.52	-----------	3.13 -3.89	56.6	3.15- 3.89	55.4
**5″**	3.38 - 3.80	80.9	3.83 – 3.52	-----------	3.13 -3.89	81.3	3.15- 3.89	60.8
**6″**	3.38 - 3.80	59.8	3.83 – 3.52	-----------	3.13 -3.89	82.0	3.15- 3.89	69.8
**1‴**	4.78 [1H, d, (9.5)]	74.2	-----------	-----------	-----------	-----------	-----------	-----------
**2‴**	3.38 - 3.80	71.9	-----------	-----------	-----------	-----------	-----------	-----------
**3‴**	3.38 - 3.80	77.9	-----------	-----------	-----------	-----------	-----------	-----------
**4‴**	3.38 - 3.80	70.6	-----------	-----------	-----------	-----------	-----------	-----------
**5‴**	3.38 - 3.80	81.9	-----------	-----------	-----------	-----------	-----------	-----------
**6‴**	3.38 - 3.80	61.3	-----------	-----------	-----------	-----------	-----------	-----------

Chemical shifts (δ) are in ppm and coupling constants (J em Hz) are given in parentheses.

Compound 2 also exhibited a UV spectrum (λmax = 332, 271 nm, Figure 3C2) that was characteristic of a flavone. In addition, it exhibited a [M-H]^−^ at *m/z* 563 and MS^2^ ions at *m/z* 503([^0,3^X_b_]^−^; [M-H]^−^ -60), *m/z* 473 ([^0,3^X_a_]^−^; [M-H]^−^ -90), *m/z* 383 ([^0,3^X_a a_nd ^0,2^X_b_]^−^; [M-H]^−^ -180), *m/z* 353([^0,2^X_a a_nd ^0,2^X_b_]^−^; [M-H]^−^ -180) (Figure 4B). This compound was identified as 6-*C*-β-*D*-glucopyranosyl-8-*C*-β-*D*-xylopyranosyl-4′,5,7-trihydroxyflavone, which is commonly known as vicenin-1(C_26_H_28_O_14_) [36].

Compound 3 (λmax = 269, 235 nm, Figure 3C3) was isolated as yellow crystals and exhibited a [M-H]^−^ at *m/z* 431 and MS^2^ ions at *m/z* 311 ([^0,2^X]^−^; [M-H]^−^ -120), and *m/z* 341 ([^0,3^X]^−^; [M-H]^−^ -90), (Figure 4C). The ^1^H NMR spectrum (Table 1) showed resonances for two sets of doublets at δH 8.02 and 6.89, which were assigned to H-2′, 6′ and H-3, respectively. In addition, two singlets at δ 6.77 and δ 6.26 were assigned to H-3 and H-6, respectively. These data indicated that the aglycone moiety was analogous to apigenin. The broad singlet at δ 13.16 was attributed to the -OH group, which forms hydrogen bonds. The presence of an anomeric proton at δ 6.2 suggested that this compound is a C8-glucosil flavone [37, 38]. Comparing the spectroscopic data of compound 3 with the reported ones confirmed its structure to be 5, 7, 4′-trihydroxyflavone- 8-C-β-D-glucopyranoside, which is commonly known as vitexin (C_21_H_20_O_10_) [37, 38].

Compound 4 (λmax = 335, 271 nm, Figure 3C4) was isolated as yellow crystals (Figure 3C4). The quasimolecular ion [M-H]^−^ was observed at *m/z* 431 and its MS^2^ fragments at *m/z* 413 ([M-H]^−^ -18), *m/z* 341 ([^0,3^X]^−^; [M-H]^−^ -90) and *m/z* 311 ([^0,2^X ]^−^; [M-H]^−^ -120) are similar to compound 3 (Figure 4D). The ^1^H NMR spectrum (Table 1) of this compound showed one singlet at δ 6.75, which was attributable to H-3, two signals corresponding to the ring B at δ 6.92 (d, J = 9.0 Hz and 3.0 Hz; H-5′ and H-3′) and 7.91 (d, J = 9.5 Hz; H-2′ and H-6′) indicating the presence of a 4′-OH group. The broad singlet at δ 13.54 was attributed to the -OH groups, which form hydrogen bonds. The signal at δ 6.26 (singlet) was attributed to H-8 of ring A, which is characteristic of a C-glycoside. The anomeric proton signals for the isovitexin appeared at δ 4.69 (d, J = 9.5 Hz; H-1″). The presence of an anomeric proton at δ 4.69 (d, J = 9.5 Hz; H-1″) attached to carbon at δ73.66 suggested that compound 4 is a C-glucoside flavone. The sites of the sugar linkage to the aglycone were considered to be at the C-6 positions, due to the presence of a quaternary C-6- signal at δ 109.29 and a protonated C-8 signal at δ94.32. Comparing the spectroscopic data of compound 3 with published spectra [39–41] confirmed its structure to be 5,7,4′-trihydroxyflavone- 6-C-β-D-glucopyranoside, which is commonly known as isovitexin (C_21_H_20_O_10_).

Compound 5 (λmax = 342, 270 nm, Figure 3C5) exhibited a [M-H]^−^ ion at *m/z* 461 and MS^2^ ions at *m/z* 371 ([^0,3^X]^−^; [M-H]^−^ -90), *m/z* 383 ([^0,4^X]^−^; [M-H]^−^ -60-18), *m/z* 341 ([^0,2^X]^−^; [M-H]^−^ -120) (Figure 4E). This compound was isolated as white crystals, and its ^1^H NMR spectrum (Table 1) included signals at δ 6.75 (singlet) due to H-3, signals at δ 6.92 (d, J = 8.0Hz) for H-3′/H-5′, δ 7.92 (d, J = 8.0Hz) for H-2′/H-6′, and the presence of the OMe, which was confirmed by a singlet at δ 3.89 (4H’). The broad singlet at δ 13.54 was attributed to the -OH groups, which form hydrogen bonds. The presence of an anomeric proton at δ 4.59 (d, J = 9.5 Hz; H-1″) attached to carbon at δ 123.06 suggested that compound 4 is C-glucoside flavone. The sugar linkages to the aglycone were considered to be at the C-6 positions, due to the presence of a quaternary C-6- signal at δ 93.4. Comparing the spectroscopic data of compound 5 with published spectra [42] confirmed its identity as 6-C-glycoside-diosmetin (C_22_H_20_O_10_).

Compound 6 (λmax = 305, 265 nm, Figure 3C6) exhibited a [M-H]^−^ at *m/z* 269 and MS^2^ ions at *m/z* 225 ([^0,4^X]^−^; [M-H]^−^ -46) and *m/z* 241([^0,4^X]^−^; [M-H]^−^ -28) (Figure 4F). This compound was identified as 4′, 5, 7-trihydroxyflavone, which is commonly known as apigenin (C_15_H_10_O_5_).

Analysis of the data obtained by HPLC-ESI-MS^n^ revealed that vitexin, isovitexin and 6-C-glycoside-diosmetin are present within the flavonoidic fraction FfA, and furthermore, that all of the flavones identified were present in the FfB.

The identification of vitexin and isovitexin was supported by the co-injection of the standards and CE. The isolated substances (i.e., vicenin-2, vitexin, isovitexin and 6-C-glycoside-diosmetin) were quantified by HPLC and yours content in the crude extract CE were 0.47 ± 0.24; 0.29 ± 0.15; 0.25 ± 0.06 and 0.20 ± 0.06 (mg/g ± SD).

### The Crude Extract of E. *falcata*(CE) and Flavonoidic Fractions improves the acquisition of fear memory

**Figure 5:**
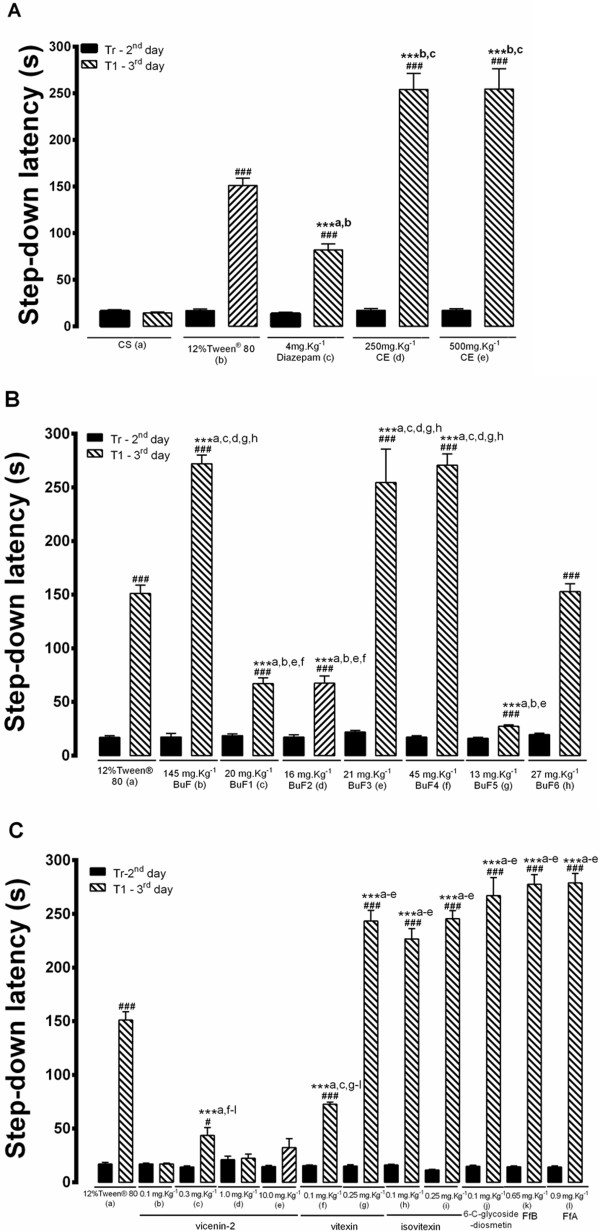
**A graph showing the effects of acute treatment with the crude extract of** ***Erythrina falcata*** **(CE) (A), buthanolic fraction (BuF), the subtraction from BuF (B) and the flavonoidic fraction (FfA and FfB) (C), as well as the negative (CS, Tween) and positive control (Diazepam) treatments, on the acquisition of fear memory as evaluated by one-trial, step-down inhibitory avoidance (IA).** The values for step-down latency are expressed as the mean values (±SEM). *P < 0.05, **P < 0.01 and ***P < 0.001, according to ANOVA followed by the Bonferroni’s Multiple Comparison Test. ^**###**^ *P* < 0.001, comparasion between Training and Test, according to ANOVA followed by the Bonferroni’s Multiple Comparison Test. The drugs and vehicle were administered at 30 min prior to the training sessions using the flavonoidic fractions (FfA and FfB). No *drugs were administered* during the test.

The effect of CE, (250 mg.Kg^−1^and 500 mg.Kg^−1^), and the control treatment (4 mg.Kg^−1^ Diazepam and Tween® 80, n = 10/group) on inhibitory avoidance memory retention are shown in Figure 5A. The differences in mean values (SEM) of step-down latencies between the Tr and T1 sessions were considered as a measure of retention of fear memory. No significant differences were observed among the groups in terms of step-down latencies of the training session (*P* > 0.05). There was a significant difference in step-down latencies between the groups (control × versus treated group) in the retention test session [*F*_(11,108)_ = 112.5, *P* < 0.0001], with the exception of the CS group, in which no differences were observed in the training session step-down latency values (*P* > 0.05). Comparisons between the training and test session step-down latencies revealed an effect on the acquisition of memory for the controls and CE groups (*P* < 0.0001), with the exception of the CS group (*P* > 0.05). The analyses of the mean step-down latency values for each group in the T1 period revealed that the CE-treated rats, at both doses, exhibited an increase in mean latency compared to the CS group (*P* < 0.0001), Tween® group (*P* < 0.0001) and Diazepam (*P* < 0.0001). Rats treated with Diazepam exhibited reduced latencies compared to the Tween® group (*P* < 0.0001). In summary, treatment with CE improved the acquisition of fear memory during the T1 session. Conversely, Diazepam decreased the retention latency values of the T1 session compared to the Tween® and CE groups.

The mean step-down latency values of the Tr and T1 session animals treated with 145 mg.Kg^−1^BuF, subfractions of BuF (20 mg.Kg^−1^ BuF1, 16 mg.Kg^−1^ BuF2, 21 mg.Kg^−1^ BuF3, 45 mg.Kg^−1^ BuF4, 13 mg.Kg^−1^ BuF5 or 27 mg.Kg^−1^ BuF6) and the values of the corresponding control animals (Tween®) prior to the training session are shown in Figure 5B. There were no significant differences among the groups in terms of step-down latencies of the training session (*P* > 0.05) according to one-way ANOVA. A significant difference was observed between the groups (Tween® × treated group) of the T1 session [*F*_(15,139)_ = 110.3, *P* < 0.0001]. Comparisons between the training and test sessions revealed an effect on the acquisition of memory for the groups treated with BuF and the subfractions (*P* < 0.0001), except for the BuF5-treated group (*P* > 0.05). Analyses of groups in the T1 session revealed a significant increase in mean step-down latencies for the groups treated with 145 mg.Kg^−1^BuF, 21 mg.Kg^−1^BuF3 or 45 mg.Kg^−1^BuF4 compared to those treated with Tween®, BuF1, BuF2, BuF5 and BuF6 (*P* < 0.0001). Rats treated with 20 mg.Kg^−1^BuF1, 16 mg.Kg^−1^BuF2 and 27 mg.Kg^−1^BuF5, exhibited significantly shorter step-down latencies than those treated with Tween®, BuF, BuF3 and BuF4 (*P* < 0.0001). No differences in values were observed in the BuF6 and Tween® groups (*P* > 0.05).

In summary, treatment with 145 mg.Kg^−1^BuF, 21 mg.Kg^−1^BuF3 and 45 mg.Kg^−1^ BuF4 improved the acquisition of conditioned fear.

Shown in Figure 5C are the step-down latency values of the animals treated with vicenin-2 (0.1, 0.3, 1.0 or 10.0 mg.Kg^−1^), vitexin (0.1 or 0.25 mg.Kg^−1^), isovitexin (0.1 mg.Kg^−1^ or 0.25 mg.Kg^−1^), 0.1 mg.Kg^−1^ 6-C-glycoside-diosmetin, 0.65 mg.Kg^−1^ FfB or 0.9 mg.Kg^−1^ FfA in the Training (Tr) and Test (T1) sessions. There were no significant differences among the groups in terms step-down latencies in the training session (*P* > 0.05), as indicated by One-way ANOVA. Comparisons of both training and test sessions revealed a significant effect on step-down latencies in terms of the acquisition of memory for the groups treated with Tween®, vitexin, isovitexin, 6-C-glycoside-diosmetin, FfA, FfB (*P* < 0.0001) or 0.3 mg.Kg^−1^ vicenin-2 (*P* < 0.05). No differences were observed in the groups treated with vicenin-2 (0.1, 1.0 or 10 mg.Kg^−1^) upon comparison of the Tr and T1 sessions. One-way ANOVA revealed a significant effect on all of the groups treated in the T1 session [*F*_(24,225)_ = 296.4, *P* < 0.0001]. The retention of step-down latencies of the groups treated with 0.25 mg.Kg^−1^ vitexin, isovitexin, 6-C-glycoside-diosmetin, FfA or FfB were significantly increased compared to those of Tween® and vicenin-2 and 0.1 mg.Kg^−1^ vitexin (*P* < 0.0001) in T1 session. The rats treated with 0.3 mg.Kg^−1^ vicenin-2 and 0.1 mg.Kg^−1^ vitexin (*P* < 0.0001) exhibited significantly reduced step-down latencies compared to those treated with Tween® and 0.25 mg.Kg^−1^ vitexin, isovitexin, 6-C-glycoside-diosmetin, FfA or FfB. In summary, treatment with flavonoidic fractions, 0.25 mg.Kg^−1^ vitexin, isovitexin and 6-C-glycoside-diosmetin improved the acquisition of fear memory in the T1 session. Conversely, treatment with 0.3 mg.Kg^−1^ vicenin-2 and 0.1 mg.Kg^−1^ vitexin decreased retention latency in the T1 session compared to treatment with Tween® and flavonoidic fractions, 0.25 mg.Kg^−1^ vitexin, isovitexin and 6-C-glycoside-diosmetin groups. Nevertheless, the rats subjected to treatments with vicenin-2 (0.1, 1.0 or 10.0 mg.Kg^−1^) exhibited impaired fear memory when evaluated in T1.

### Treatment with the Crude Extract of E. *falcata*(CE) or Flavonoidic Fractions resulted in significant retention of fear memory without impairing the extinction of fear memory

**Figure 6:**
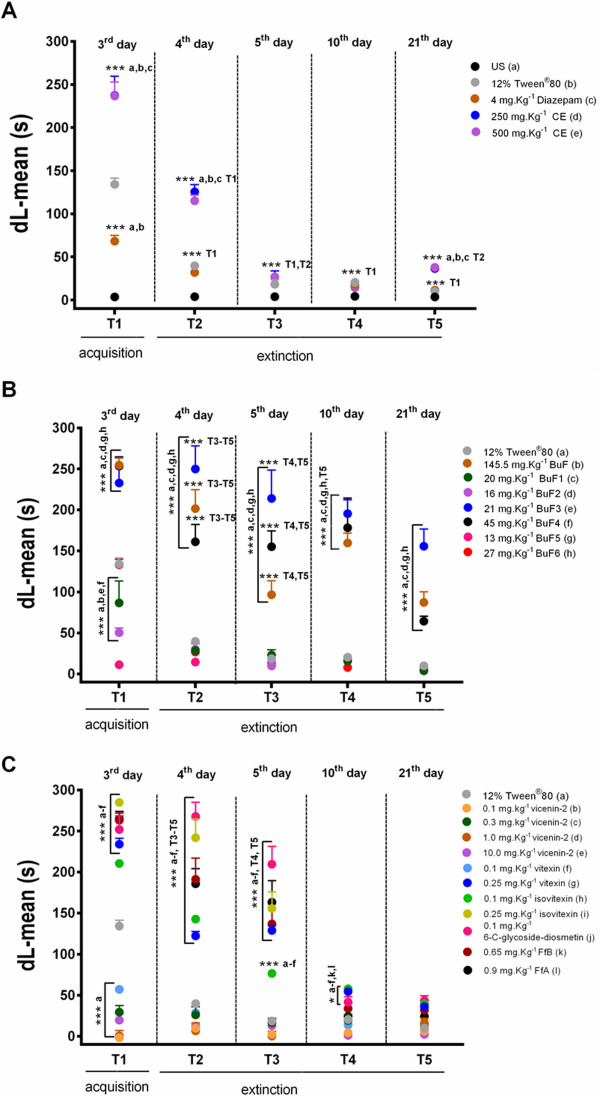
**A graph showing the effects of acute treatment with the crude extract of** ***Erythrina falcate*** **(CE) (A), buthanolic fraction (BUF), subfraction from BuF (B) and the flavonoidic fractions (FfA and FfB) (C) on the acquisition and extinction of fear memory as evaluated by one-trial, step-down inhibitory avoidance (IA).** The animals were subjected to five retention sessions (T1-T5) of IA tasks. The values of the step-down latencies are expressed as the as mean differences of the latencies between Training and Test sessions (dL = mean ± EPM). *P < 0.05, **P < 0.01 and ***P < 0.001. No footshock was administered during the test sessions. The interval between T1, T2 and T3 was 24 h. T4 and T5 comprised 240- and 480-h intervals, respectively, after training. No drugs were administered during the test sessions. Two-way ANOVA was used to consider the two fixed factors (group and trial) and one random factor (rat). Repeated measures were employed for the intragroup comparison of the retention test data (CS presentation).

Shown in Figure 6A are the values for retention of fear memory as evaluated by the dL-mean (Test-Tr latency) test of the groups treated with Tween®, 4 mg.Kg^−1^ Diazepam and CE (250 mg.Kg^−1^ or 500 mg.Kg^−1^) in the acquisition (T1) and extinction sessions (T2-T5). Two-way ANOVA revealed significant effects of the treatments [*F* (5,54) = 102.8, *P* < 0.0001], sessions [*F* (4,216) = 276.7, *P* < 0.0001] and significant treatment × session interaction [*F* (20,216) = 27.20, *P* < 0.0001]. Analysis of the groups on the 4th day (T2) revealed significant differences in terms of the dL-mean for group treated with 250 mg.Kg^−1^ CE (dL = 116.22 ± 6.93, *P* < 0.001) or 500 mg.Kg^−1^ CE (dL = 127.33 ± 8.48, *P* < 0.001) when compared to those treated with Tween® (dL = 39.7 ± 4.83), CS (dL = 3.9 ± 0.80) or Diazepam (dL = 32.0 ± 4.64) groups. No differences were observed when the Diazepam group was compared to the Tween® group (*P* > 0.05), but we had observed differences in relation to CS group (*P* < 0.0001). On the 5th day (T3), no significant differences were observed in terms of dL-mean values for the groups treated with Diazepam (dL = 18.30 ± 2.79, *P* > 0.05), 250 mg.Kg^−1^ CE (dL = 28.55 ± 4.15, *P* > 0.05) or 500 mg.Kg^−1^ CE (dL = 29.11 ± 6.91, *P* > 0.05) compared to the control-treated groups (Tween®, dL = 18.5 ± 5.04 and CS, dL = 3.8 ± 0.54). The analysis of step-down latency on the 10th day after training (T4) revealed that all of the groups exhibited mean dL values of approximately 15 s (Diazepam, dL = 17.6 ± 2.13; 250 mg.Kg^−1^ CE; DL = 13.55 ± 6.00, 500 mg.Kg^−1^ CE dL = 16.44 ± 4.55), except CS group (dL = 4.3 ± 0.95 (*P* < 0.001). Here, no differences were observed among the groups (*P* > 0.05). All of the animals tested on the 21st day after training (T5) exhibited extinction of fear memory as evaluated in IA task, with the exception of the CS group, which did not acquire fear-conditioned memory. Differences were observed among the groups treated with 250 mg.Kg^−1^ CE (dL = 38.7 ± 3.16) or 500 mg.Kg^−1^ CE (dL = 36.44 ± 2.21) compared to those treated with Diazepam (dL = 11.5 ± 2.16), CS (dL = 3.6 ± 1.24) or Tween® (dL = 15.5 ± 3.04) (*P* < 0.001). Intragroup comparisons revealed that all of the groups exhibited significantly decreased dL-mean values in the T2 session compared to the T1 session (P < 0.0001). Rats treated with the both doses of CE exhibited spontaneous recoveries in the T2 session and decreased dL-mean values in the T3 - T5 sessions compared to the T2 (*P* < 0.0001) and T1 (*P* < 0.0001) sessions. No differences were observed in dL-mean values of the Tween®, Diazepam and CS-treated groups in the T3 - T5 sessions (*P* > 0.05). In summary, every group acquired conditioned fear. Nonetheless, the CE groups exhibited significant dL-mean values during the T2 extinction session.

Shown in Figure 6B are values of retention of fear memory as evaluated by the dL-mean test of the groups treated with of 145 mg. Kg^−1^BuF, subfractions of BuF (20 mg.Kg^−1^BuF1, 16 mg.Kg^−1^BuF2, 21 mg.Kg^−1^BuF3, 45 mg.Kg^−1^ BuF4, 13 mg.Kg^−1^BuF5 or 27 mg.Kg^−1^BuF6) and Tween® in the acquisition (T1) and extinction sessions (T2-T5). Two-way ANOVA revealed a significant effect of treatment [*F* (7,63) = 72.44, *P* < 0.0001], sessions [*F* (4,36) = 71.56, *P* < 0.0001] and of significant interaction of treatment x sessions [*F* (28,252) = 5.475, *P* < 0.0001]. Significant differences in the dL-mean values were observed on the 4th day (T2) for the groups treated with BuF (dL = 201.96 ± 22.92, *P* < 0.0001), BuF3 (dL = 278 ± 1.45), BuF4 (dL = 161.1 ± 21.23, *P* < 0.0001) or BuF6 (dL = 112.5 ± 24.80, *P* < 0.001) compared to the group treated with Tween® (dL = 39.7 ± 4.83). However, no differences were observed among the groups treated with BuF1 (dL = 34.5 ± 11.94, *P* > 0.05), BuF2 (dL = 30.1 ± 6.71, *P* > 0.05), or BuF5 (dL = 14.4 ± 2.31, *P* > 0.05). On the 5th day (T3), there were significant differences in the long-term memory retention of the group treated with Tween® (dL = 18.5 ± 5.04) at 72 h after training compared to the groups treated with BuF (dL-mean: 96.6.1 ± 16.98, *P* < 0.0001), BuF3 (dL = 239.77 ± 24.58, *P* < 0.0001) of BuF4 (dL = 155.2 ± 19.17, *P* < 0.0001), which exhibited increased step-down latencies. However, no significant differences were observed in the groups treated with BuF1 (dL = 21.75 ± 6.76, *P* > 0.05), BuF2 (dL = 9.8 ± 1.97, *P* > 0.05), BuF5 (dL = 13.4 ± 4.70, *P* > 0.05) or BuF6 (dL = 28.4 ± 7.09, *P* > 0.05). These groups exhibited impaired acquisition of fear memory. The analysis of the data from the 10th day after training (T4) revealed significant differences in the dL-mean values of the groups treated with BuF (dL = 159.7 ± 11.93, *P* > 0.0001), BuF3 (dL = 199.4 ± 17.34, *P* > 0.0001), or BuF4 (dL = 178.2 ± 36.28, *P* > 0.0001). These groups exhibited increased step-down latencies compared to the groups treated with Tween® (dL = 20.5 ± 5.04), BuF1 (dL = 14.12 ± 3.87), BuF2 (dL = 19.7 ± 3.70), BuF5 (dL = 15.80 ± 3.56) and BuF6 (dL = 7.96 ± 1.71). Significant differences in the dL-mean values were observed on the 21st day (T5) in animals tested after training and treatment with BuF (dL = 87.2 ± 12.80, *P* < 0.0001), BuF3 (dL = 153.1 ± 22.01, *P* < 0.0001) or BuF4 (dL = 64.3 ± 6.05, *P* < 0.05) compared to the groups treated with Tween® (dL = 9.9 ± 1.1), BuF1 (dL = 3.62 ± 1.01, *P* > 0.05), BuF2 (dL = 6.2 ± 1.73, *P* > 0.05) or BuF6 (dL = 6.20 ± 1.93, *P* > 0.05). Treatment with BuF5 (dL = 6.90 ± 1.96) did not influence the extinction of fear memory, because the animals did not acquire fear memory. Intragroup comparisons revealed that BuF1 and BuF6 groups exhibited decreased dL-mean values in the T2-T5 sessions compared to the T1 session (*P* < 0.001). No differences in dL-mean values were observed in the groups treated with BuF5 when the T1 and T5 sessions were compared (*P* > 0.05). The groups treated with BuF, BuF3 or BuF4 exhibited decreased dL-mean values in the T2- T5 sessions compared to the T1 (*P* < 0.0001). In summary, these data showed that fear memory was not attenuated by repeated testing after treatment with BuF, BuF3 or BuF4, resulting in significantly improved retention of fear memory when tested at 48, 72, 240 and 480 hours. These groups were particularly resistant to extinction.

Shown in Figure 6C are the values for retention of fear memory as evaluated by the dL-mean test of the groups treated with Tween®, vicenin-2 (0.1, 0.3, 1.0 and 10.0 mg.Kg^−1^), vitexin (0.1 or 0.25 mg.Kg^−1^), isovitexin (0.1 or 0.25 mg.Kg^−1^), 0.1 mg.Kg^−1^ 6-C-glycoside-diosmetin, 0.65 mg.Kg^−1^ FfB and 0.9 mg.Kg^−1^ FfA. Two-way ANOVA revealed a significant effect of treatment [*F* (11,107) = 129.7, *P* < 0.0001], sessions [*F* (4,428) = 450.0, *P* < 0.0001] and the interaction of treatment x sessions [*F* (44,428) = 34.70, *P* < 0.0001]. Significant differences in the dL-mean values were observed on the 4th day (T2) for the groups treated with 0.25 mg.Kg^−1^vitexin (dL = 242 ± 22.89, *P* < 0.0001), 0.1 mg.Kg^−1^ isovitexin (dL = 142.2 ± 7.0, *P* < 0.0001), 0.25 mg.Kg^−1^ isovitexin (dL = 242.3 ± 5.29, *P* < 0.0001), 6-C-glycoside-diosmetin (dL = 267.9 ± 17.12, *P* < 0.0001), FfA (dL = 191.2 ± 25.69, *P* < 0.0001), and FfB (dL = 185.9 ± 18.28, *P* < 0.0001) compared to the groups treated with Tween® (dL = 39.7 ± 4.83), but no significant differences were observed for any of the doses of vicenin-2 (0.1, 0.3, 1.0 and 10.0 mg.Kg^−1^) and (0.1 mg.Kg^−1^vitexin (*P* > 0.05). On the 5th day (T3), there were significant differences between the group treated with 0.25 mg.Kg^−1^ vitexin (dL = 128.8 ± 20.13, *P* < 0.0001), 0.1 mg.Kg^−1^ isovitexin (dL = 76.5 ± 1.73, *P* < 0.0001), 0.25 mg.Kg^−1^ isovitexin (dL = 155.8 ± 6.08, *P* < 0.0001), 6-C-glycoside-diosmetin (dL = 209.7 ± 21.65, *P* < 0.0001), FfA (dL = 136.9 ± 6.67, *P* < 0.0001), and FfB (dL = 163.3 ± 26.21, *P* < 0.0001) compared to the groups treated with Tween® (dL = 18.5 ± 5.04), 0.1 mg.Kg^−1^vitexin (dL = 15.9 ± 2.20), 0.1 mg.Kg^−1^vicenin-2 (dL = 2.4 ± 1.8), 0.3 mg.Kg^−1^vicenin-2 (dL = 18.44 ± 5.44), 1.0 mg.Kg^−1^vicenin-2 (dL = 12.4 ± 5.40) and 10.0 mg.Kg^−1^vicenin-2 (dL = 12.8 ± 6.34), indicating increased step-down latencies. No significant differences in the dL-mean values were observed in the groups treated with vicenin-2 (0.1, 1.0, 0.3 or 10.0 mg.Kg^−1^) and 0.1 mg.Kg^−1^vitexin compared to the groups treated with Tween® (*P* > 0.05), indicating these groups exhibited reduced dL step-down latencies. The analysis of these data from the 10th day after training (T4) revealed no significant differences in dL-mean values compared to groups treated with 0.1 mg.Kg^−1^ vitexin (dL = 13.7 ± 1.2, *P* > 0.05), 0.3 mg.Kg^−1^ vicenin-2 (dL = 14.88 ± 5.92, *P* > 0.05), 1.0 mg.Kg^−1^ vicenin-2 (dL = 11.4 ± 4.63, *P* > 0.05), 10.0 mg.Kg^−1^ vicenin-2 (dL = 3.4 ± 1.54, *P* > 0.05), 0.65 mg.Kg^−1^ FfB (dL = 33.8 ± 6.67, *P* > 0.05) or 0.9 mg.Kg^−1^ FfA (dL = 24.2 ± 2.96, *P* > 0.05) when compared with Tween® (dL = 20.5 ± 1.1), but significant differences in the dL-mean values were observed in the groups treated with 0.25 mg.Kg^−1^ vitexin (dL = 54.5 ± 2.52, *P* < 0.0001), 6-C-glycoside-diosmetin (dL = 41.6 ± 6.59, *P* < 0.05), 0.1 mg.Kg^−1^ isovitexin (dL = 57.9 ± 1.79, *P* < 0.05) or 0.25 mg.Kg^−1^ isovitexin (dL = 54.5 ± 4.03, *P* < 0.05) compared to the all groups. No significant differences in the dL-mean values were observed in animals tested on the 21st day after training (T5) and treatment with 0.1 mg.Kg^−1^ vitexin (dL = 14.7 ± 1.55, *P* > 0.05), 0.25 mg.Kg^−1^ vitexin (dL = 36.1 ± 2.20, *P* > 0.05), 0.1 mg.Kg^−1^ vicenin-2 (dL = 6.3 ± 1.84, *P* > 0.05), 0.3 mg.Kg^−1^ vicenin-2 (dL = 11.55 ± 2.90, *P* > 0.05), 1.0 mg.Kg^−1^ vicenin-2 (dL = 19.8 ± 4.63, *P* > 0.05), 10.0 mg.Kg^−1^ vicenin-2 (dL = 2.2 ± 1.98, *P* > 0.05), 0.1 mg.Kg^−1^ isovitexin (dL = 39.5 ± 2.00, *P* > 0.05), 0.25 mg.Kg^−1^ isovitexin (dL = 14.7 ± 2.00, *P* > 0.05), 0.1 mg.Kg^−1^ 6-C-glycoside-diosmetin (dL = 43.5 ± 5.71, *P* > 0.05), 0.65 mg.Kg^−1^ FfB (dL = 31.7 ± 6.17, *P* > 0.05) or 0.9 mg.Kg^−1^ FfA (dL = 24.1 ± 3.08, *P* > 0.05) compare to the group treated with Tween® (dL = 9.9 ± 1.1). Intragroup comparisons revealed that rats treated with isovitexin (0.1 or 0.25 mg.Kg^−1^) exhibited a gradual decrease in the dL-mean values of the T2 and T3 sessions compared to the T1 session (*P* < 0.0001). Significant differences in the dL-mean values were observed on groups treated with 6-C-glycoside-diosmetin in the T3 session compared to the T1 and T2 (*P* < 0.0001) sessions. Rats treated with vitexin (0.1 or 0.25 mg.Kg^−1^) exhibited a decreased in dL-mean values in the T2 session compared to the T1 session (*P* < 0.0001). No differences in dL-mean values were observed in the group treated with vicenin-2 (0.1, 0.3, 1.0 or 10.0 mg.Kg^−1^) when comparing values for the T1 - T5 sessions (*P* > 0.05). In sessions T4-T5, all of the groups exhibited decreased dL-mean values when compared to the T1, T2 and T3 sessions (*P* < 0.0001), with the exception of the group treated with vicenin-2.

In summary, with the exception of the group treated with vicenin-2 (0.1, 1.0 and 10 mg.Kg^−1^) every groups exhibited the acquisition and extinction of fear memory. However, treatment with vitexin (0.1 or 0.25 mg.Kg^−1^), isovitexin (0.1 or 0.25 mg.Kg^−1^), and 0.1 mg.Kg^−1^6-C-glycoside-diosmetin, enhanced the retention of fear memory, as indicated in the T1-T3, but did not prevent the extinction of fear memory.

## Discussion

Phytochemical analysis has demonstrated that most of the secondary metabolites characterized in our study are flavones, and for the first time, are identified in the plant *Erythrina falcata*. Furthermore, monitored fractionation using a one-trial step-down inhibitory avoidance task (IA) showed that the CE, buthanolic total fraction (BuF), subfractions and flavonoidic fractions modulated the acquisition and extinction of fear memory. Rats treated with 0.25 mg.Kg^−1^ vitexin, isovitexin (0.1 or 0.25 mg.Kg^−1^) and 6-C-glycoside-diosmetin and FfA or FfB exhibited enhanced retention of fear memory, as indicated in T1 to T3. In addition, the treatment with BuF, BuF3 and BuF4, resulted in particular resistance to extinction.

### The molecular structure of the secondary metabolites of the crude extract of *E. falcata*

The use of hyphenated techniques has been employed to characterize and identify flavonoids in natural products. The power of combining separation technologies with spectroscopic techniques has been extensively demonstrated for both the quantitative and qualitative analysis of unknown compounds in complex natural product extracts or fractions. To obtain structural information for the identification of the compounds present in a crude sample, various modern hyphenated techniques have been applied, including for example, GC-MS, LC-MS, and LC-NMR. HPLC is the most widely used analytical separation technique for the qualitative and quantitative determination of compounds in natural product extracts [43]. In this study, we used HPLC-ESI/MS^n^ to structurally characterize and identify the compounds present in CE, because this technique facilitates the rapid screening of crude natural product extracts or fractions for detailed information, such as metabolic profiles, using a minimal amount of material.

Flavonoids commonly exist as flavonoid O-glycosides, in which one or more of the hydroxyl groups of the aglycone are bound to a sugar, forming a glycosidic O-C bond. However, flavonoids can also exist as flavonoid C-glycosides, in which glycosylation might occur directly through the linkage of the sugar to the basic nucleus of the flavonoid via a C-C bond [44–46].

According to our UV spectral profiles, the majority of the CE compounds exhibit a predominance of flavones. The UV spectra of flavones exhibited two strong absorption peaks that are commonly referred to as band I (300–380 nm) and band II (240–280 nm). Band I is associated with the presence of a cinnamoyl system that involves ring B, whereas Band II is attributed to an A-ring with a benzoyl system. Substitutions on rings A or B might produce hypsochromic or bathochromic shifts of the absorption peaks, which are useful for clarifying structures. In the flavone glycosides, O- and C- glycosylation of ring A appears to elicit little-to-no effect on their UV spectral profiles, but glycosylation of the ring B induces shifts of band I to lower wavelengths (hypsochromic shift), with this effect being increased for a resonant position (4′) than for a non-resonant position (3′) [33]. Flavones C-glycosides exhibit the typical UV spectra of flavones (band I and band II of similar intensities). These findings support the spectral profile of compounds 1–6. The UV spectral profiles that we obtained from the crude extract and active fractions exhibited two main absorption peaks of approximately 260 and 331 nm, which are typical of flavones [33].

Tandem mass spectrometric fragmentation (ESI-MS/MS) has been extensively used to characterize flavonoids. The combinations of both ionization modes (positive and negative) in MS1 full scan mode yields enhanced certainty in terms of determining the molecular mass. The negative ion mode provides the optimal sensitivity and results in fragmentation, making it the most suitable for inferring the molecular mass of separated flavonoids [47].

Our HPLC-ESI/MS study permitted identified most of the flavonoids, presents in CE of in the E. *falcata*. The fragmentation pathways demonstrated the presence the two flavones di-C-glycosides (compound 1 and 2), three flavones mono-C-glycosides (compound 3, 4 and 5). The fragmentation data obtained from vicenin-2 (1) at *m/z* 593[29, 47–49], vicenin-1 (2) at *m/z* 563 [36], vitexin (3) at *m/z* 431 [29, 47, 50], isovitexin (4) at *m/z* 431 [36, 48, 50], 6-C-glycoside-diosmetin (5) at *m/z* 461 [47, 49, 51, 52] and apigenin (6) at *m/z* 269 [53] are consistent with the fragmentation data for C-glycosides that have been previously described in literature. The vicenin-1 (2) and vicenin-2 (1) were found in the first time in E. *falcata*. Vicenin-2 has been described in the other *Erythrina* species including E. *indica* [54] and E. *caffra* [35]. Both compounds were found in the *Passiflora incarnata* [55]. Vitexin and isovitexin have been described in other *Erythrina* species such as E. *indica* [54] and E. *caffra* [35]. This study, however, is the first report of such compounds in E. *falcata*. In addition, these compounds can also be found as active components of extracts derived from *Passiflora incarnata* [55]. Notably, this is the first description of 6-C-glycoside-diosmetin in E. *falcata* plants and in the *Erythrina* genus.

For the first time, our results demonstrated that predominant secondary metabolites found in crude dry extracts of *Erythrina falcata* belong to a subclass of flavone C-glycosides and might represent a potential marker of this extract. According to our quantitative analyses vicenin-2, vitexin and isovitexin represent the most abundant constituents, whereas conversely, 6-C-glycoside-diosmetin was identified as the least abundant constituent. Furthermore, our bioactivity-guided study demonstrated that these compounds, which were present in both flavonoidic fractions, are attributable for the biological effects observed in this study.

### Monitored fractionation study in the acquisition and extinction of fear memory

Our main finding was that treatment with CE, BuF, BuF3 and BuF4, vitexin, isovitexin, 6-C-glycoside-diosmetin and flavonoidic fractions (FfA and FfB) can improve the acquisition of fear memory. These flavone mono-C-glycosides, both doses of isovitexin, 0.25 mg.Kg^−1^ vitexin, 6-C-glycoside-diosmetin and both flavonoidic fractions induced retention of extinction of fear. Furthermore, rats treated with BuF, BuF3 and BuF4 were resistant to extinction. Together, these findings suggest that components present in the butanolic fraction (BuF), also found in the subfractions 3 and 4 (BuF3 and BuF4), can modulate the fear extinction. This hypothesis is established in data from treatments with CE or isolated flavones mono-C-glycosides, which acquired the fear extinction memory. In this sense, the interaction among compounds presents in the BuF as well as subfractions BuF3 and BuF4 can modulate fear memory acquisition and possibly their consolidation as well. This raises the question of the adaptive value of flavones in memory formation. To better understand our findings, a current description concerning the role of flavonoids in memory formation is required.

Converging evidence over the last few decades has shown that extracts of flavonoid-rich plant or flavonoid molecules have been the focus of numerous researches as potent modulators of memory formation and cognition. Recent evidence has indicated that the consumption of flavonoid-rich plants or/and foods, extracts, and purified flavonoids are correlated with improved memory, which prevents age-related spatial learning deficit in mice [56] or improves fear memory acquisition [2]. Additionally, it has been suggested that improved memory flavonoid-induced seems attributable to their ability in modulating different neurotransmitter systems.

Generally, flavonoids act as modulators of the GABA_A_ receptors [57–59]. However, they can also modulate others receptors that are important for neural plasticity (e.g., TrKB and NMDA receptors) [60–62]. One way they act is by regulating proteins such as mitogen-activated protein kinase (MAPK), phosphoinositide 3-kinase (PI3 kinase)/Akt signalling cascade [22] and cAMP response element-binding protein (CREB) [1, 2].

The ability of various flavones to modulate the GABA_A_ receptor is related with a pharmacophore model that was previously proposed by Cook and Dekermendjian [63]. The “diazepam-like” effects justify the use of diazepam as a positive control in our work.

The pre-training administration benzodiazepine agonist resulted in impaired acquisition of fear memory in behavioral models such as inhibitory avoidance. Ours results, in combination with other published studies, confirms that treatment with diazepam impairs retention test performance in conditioned fear tasks.

Likewise, treatment with flavone di-C-glycoside vicenin-2, (0.1, 1.0 or 10.0 mg.Kg^−1^), impairs acquisition of conditioned fear. Conversely, rats treated with 0.3 mg.Kg^−1^ vicenin-2 and 0.1 mg.Kg^−1^ vitexin had acquisition of fear memory, but they exhibited slower latency retention in the IA tasks. These data suggest that the flavones from CE in IA may modulate different neurochemical systems.

Interestingly, the flavone mono-C-glycoside identified in our study is of the same class as 6, 2′dihydroxyflavone (DHF). A functional electrophysiological study has previously shown that DHF decreased GABA-induced currents in α_1_β_3_γ_2_, α_2_β_3_γ_2_ and α_5_β_3_γ_2_ subunits but not in the α_3_β_3_γ_2_ subunit of the GABA_A_ receptors [64]. The authors also demonstrated that DHF enhanced cognitive performance in the step-through passive avoidance test and elicited an anxiogenic-like effect when evaluated in the elevated plus-maze test, thus functioning as a partial inverse agonist-like modulator of the GABA_A_ receptor [64]. Thus, we hypothesize that flavone mono-C-glycosides, as well as FfA and FfB might function as partial inverse agonist-like modulators of the GABA_A_ receptor. However, further studies are required to determine of action of these bioactive molecules on GABA_A_ receptor and to understand their molecular and functional action as modulators of acquisition and extinction of fear memory.

FfA contains three flavone mono-C-glycosides (vitexin, isovitexin and 6-C-glycoside-diosmetin) and FfB contains all of the flavonoids identified in this study. Both of the flavonoidic fractions improved the acquisition fear memory, consistent with our findings of improved fear conditioned memory in short-term [2] and long-term [1] treatment groups that were administered a standardized, flavonoid-rich extract of *Ginkgo biloba* (EGb). Other studies have demonstrated beneficial effects of *Ginkgo biloba* extract on memory [65–67]. A beneficial effect of flavonoid-rich blueberry supplements on the acquisition of spatial memory has also been described [18]. These findings, in combination with our data regarding treatment with the flavonoidic fractions, confirm the beneficial effects elicited on fear memory. Thus, the presence of vicenin-2 in FfB and CE did not impair retention test performance in the conditioned fear tasks.

Repeated testing for inhibitory avoidance every 24 h after training resulted in significant retention of fear memory in the groups treated with CE, vitexin, isovitexin and 6-C-glycoside-diosmetin or both flavonoidic fractions. However, rats treated with BuF, BuF3 or BuF4 were particularly resistant to extinction.

Substances that facilitate GABA transmission have been shown to interfere with the acquisition and consolidation of fear memory, which can be blocked by extinction when administered prior to the extinction training [68]. This evidence suggests that increasing GABA transmission can impair extinction retention, as observed with the BuF, BuF3 and BuF4 treatments. Conversely, the administration of FG7142 (N-methyl-β-carboline-3-carboxamide), a nonselective inverse GABA_A_ agonist, to rats prior to extinction training of contextual fear memory, delayed the extinction and increased the levels of freezing across the course of the session test compared with the control treatment [69]. This inverse agonist decreased GABA transmission when administered prior to extinction training and impaired extinction retention [69]. Taken together, we infer that substances that are inverse agonists of the GABA receptor do not cause retention extinction, but rather, increased the retention of memory by 72 h after training. This effect was elicited by all of the mono-C-glycosides flavones, FfA and FfB.

Extinction is regarded as a form of new learning that involves the formation of a new association between the CS and non-US. The fear responses exhibited in the presence of CS are reduced (i.e., extinguished) over the course of exposure to CS alone (within- session extinction) [68]. This reduced response was not observed for BuF, BuF3 or BuF4, indicating that these compounds prevent extinction.

Despite the evidence from our studies, few studies have examined the effects of flavonoid-rich extracts and pure flavonoids on the extinction of fear memory. For example, Andero et al. [70] demonstrated that 7,8-DHF might represent an excellent reagent for elucidating the effects of TrkB activation in learning and memory paradigms and might enhance extinction in wild-type mice. That is, 7,8-DHF can “rescue” a deficit in the extinction of conditioned fear found in animals exhibiting a prior history of a single traumatic stress exposure [70]. The authors concluded that 7,8-DHF might potentially be used for the treatment of reversing learning and extinction deficits associated with psychopathology, and furthermore, might serve as a useful adjuvant treatment for anxiety disorders that are responsive to behavioral treatments using the extinction process.

These studies sustain our hypothesis that acute treatment with flavones (vitexin, isovitexin and 6-C-glycoside-diosmetin) can elicit effects similar to those of partial inverse agonists of GABA_A_ receptors and can enhance cognitive performance in IA acquisition, without causing the cognitive deficit associated with the administration of benzodiazepines. In addition, according with our chemical data, these flavones are also found in the flavonoidic fractions (FfA and FfB) and CE which improved fear memory. Our data suggest that vitexin, isovitexin, 6-C-glycoside-diosmetin and flavonoidic fractions (FfA and FfB) might serve as a useful adjuvant treatment for memory deficits.

## Conclusions

The most important contribution of this work is the seminal characterization and identification of the compounds derived from *E. falcata* plants. Our study has revealed that this plant is rich source of flavones. In addition, our monitored fractionation study provides strong evidence for the pharmacological properties of pure flavones including vitexin, isovitexin and 6-C-glycoside-diosmetin, which can enhance the acquisition of fear memory without preventing its extinction. These flavonoids might be responsible for the effects elicited CE, FfA and FfB. However, further studies are necessary to determine the mode of action of the flavonoidic fractions and the purified flavones (vitexin, isovitexin and diosmetin-6-C-glucoside) in fear memory. Finally, these data suggest that isolated flavones or flavonoidic fractions can provide novel therapeutic approaches that might be used for the treatment of cognitive deficits.

## Abbreviations

CE: Crude extract of *Erythrina falcata*
HPLC-DAD-ESI/MS^n^: High performance liquid chromatography combined with a diode array UV detector and electrospray ionization tandem mass spectrometry
HPLC-DAD: High performance liquid chromatography with diode array UV detector
NMR: Nuclear magnetic resonance
IA: Inhibitory avoidance task
BuF: Buthanolic fraction
BuF (1–6): Buthanolic subfractions
FfA: Flavonoidic fraction A
FfB: Flavonoidic fraction B
